# Frequency of etiological agents of acute bacterial meningitis using culture and polymerase chain reaction assay

**DOI:** 10.1016/j.nmni.2021.100930

**Published:** 2021-08-10

**Authors:** H. Zeighami, S. Roudashti, Sh. Bahari, F. Haghi, N. Hesami

**Affiliations:** 1) Department of Microbiology, School of Medicine, Zanjan University of Medical Sciences, Zanjan, Iran; 2) Department of Microbiology, Zanjan Branch, Islamic Azad University, Zanjan, Iran

**Keywords:** Bacterial meningitis, cerebrospinal fluid, culture, PCR, *Streptococcus pneumonia*

## Abstract

Bacterial meningitis is one of the most severe infectious diseases with high rate of morbidity and mortality in developing countries. The current study aimed to investigate the frequency of etiological agents of bacterial meningitis among patients admitted to three hospitals in Zanjan, Iran. A total of 100 cerebrospinal fluid (CSF) samples were collected aseptically, and cytochemical analysis, Gram staining, culture, and PCR were performed. Forty-six percent of CSF samples had positive bacterial culture results. However, PCR showed a higher detection rate of bacterial meningitis causing pathogens when compared with culture (52% vs. 46%; p > 0.05). Fifty-two percent of patients with bacterial meningitis were aged <1 year. The most prevalent pathogen was *Streptococcus pneumoniae* (36.5%), followed by *Neisseria meningitidis* (28.8%) and *Streptococcus agalactiae* (15.4%). *Listeria monocytogenes* was not isolated from CSF culture. The frequency of *Haemophilus influenzae, L. monocytogenes* and *Escherichia coli* was 7.7%, 1.9% and 9.6%, respectively. Although in patients aged <1 year, *S. pneumonia, N. meningitidis* and group B streptococcus were the most common pathogens causing meningitis, and in patients aged between 1 and 10 years, *Escherichia coli* was the most common. According to the results, the culture was less effective for diagnosis of bacterial meningitis than PCR. Our findings indicate that the most common causative agents of bacterial meningitis in Iran may be vaccine-preventable pathogens. Therefore, the prevention and control measures should be considered to reduce the incidence of bacterial meningitis.

## Introduction

Bacterial meningitis is one of the most severe infectious diseases with high rate of morbidity and mortality in developing countries [[1], [2], [3]]. In 2019, worldwide meningitis mortality was more than 236 000, with approximately 2.5 million new cases [3]. Among the etiological agents of bacterial meningitis in the United States, Europe and many other developed countries, *Neisseria meningitidis, Streptococcus pneumoniae, Haemophilus influenzae* type b, *Streptococcus agalactiae* and *Listeria monocytogenes* are important agents mostly in children aged <5 years [1]. Delays in diagnosis and treatment of bacterial meningitis may contribute to its high morbidity and mortality [4]. To our knowledge, there is no sufficient data regarding the epidemiology of bacterial meningitis in Iran. Most studies have mainly focused on single meningitis causative agent and specific geographic area, while a well nationwide surveillance system was not established. In a meta-analysis conducted by Houri et al., *S. pneumoniae* (30%), *H. influenza* type b (15%), coagulase-negative staphylococci (14%) and *N. meningitidis* (13%) were the most common causes of acute bacterial meningitis in Iran [17]. On the other hand, because of the lack of a routine vaccination programme against meningeal pathogens in Iran, acute bacterial meningitis is considered as a serious problem in health care settings with high rate of morbidity and mortality [17].

Rapid detection of bacterial meningitis and appropriate antimicrobial therapy are critical to minimize neurological sequelae such as hearing loss, mental retardation, seizures and behavioural changes [5].

Cerebrospinal fluid (CSF) culture is considered as a diagnostic gold standard for acute bacterial meningitis, yet is only positive in 70–85% of patients who have not received antimicrobial therapy before lumbar puncture. Final results of the culture are often not available for ≥48 h. Furthermore, the positive rate of CSF culture is relatively low because of suboptimal storage and transportation conditions and culture practice. However, culture should be kept as the gold standard as cultured bacteria are sources of data for antibiotic susceptibility, complete subtyping, the expression of antigens that are to be included in future vaccines, and pathophysiology of isolates. Gram stain is more rapid and has good specificity, but sensitivity is poor (10–93% depending on the organism and whether or not antibiotics were given before CSF collection) [6].

PCR-based assays of CSF have been suggested as a rapid, sensitive, direct and specific diagnostic test for bacterial meningitis. This assay acts independently of bacterial growth, and detection of small amounts of pathogen DNA from non-viable bacteria could potentially facilitate diagnosis in culture-negative cases [3]. Universal PCR based on the amplification of conserved regions of 16S rRNA gene has been used for detecting and differentiating of broad range of bacterial agents of meningitis. However, the performance of 16S rRNA PCR varies in different studies, probably because of different methodologies [6]. Recently, the FilmArray Meningitis/Encephalitis Panel (BioFire Diagnostics, Salt Lake City, UT), which is a multiplexed molecular panel for the simultaneous and rapid detection of 14 pathogens (including *E. coli* K1, *H. influenzae*, *L. monocytogenes*, *N. meningitidis*, *S. agalactiae*, *S. pneumoniae*, cytomegalovirus, enterovirus, herpes simplex virus 1 and 2, human herpesvirus 6, human parechovirus, varicella-zoster virus and *Cryptococcus neoformans/C. gattii*) directly from CSF specimens demonstrated a sensitivity or positive percentage of agreement of 100% for 9 of 14 analytes [9,10].

The objective of the present study was to investigate the frequency of major bacterial pathogens of meningitis in Zanjan, Iran, including *L. monocytogenes, S. pneumoniae*, *H. influenzae*, *N. meningitidis, E. coli* and *S. agalactiae* in CSF samples using culture and PCR assay.

## Materials and methods

### Sample collection and bacterial isolation

Between March 2016 and February 2017, a total of 100 CSF samples were collected from suspected cases of bacterial meningitis admitted to three major university hospitals in Zanjan, Iran. This study was approved by the Research Ethics Committee of Zanjan University of Medical Sciences (ZUMS.REC.1393.209), and informed consent was obtained from participants. For children, the consent was obtained from parents/guardians. Criteria for bacterial meningitis and ‘confirmed’ bacterial meningitis are summarized in Table 1.

**Table 1:** CSF and Patients criteria

CSF and patient's clinical and inclusion criteria		Ref
Criteria for bacterial meningitis	Fever >38^°^C of duration <21 days	[7,8]
	Headache	
	One of the following signs: neck stiffness, altered consciousness or other meningeal signs	
‘Confirmed’ bacterial meningitis according to WHO case definition criteria	Patient presenting with clinical symptoms of meningitis and identification of a bacterial pathogen by culture or PCR in the CSF sample.	[8]
Patients inclusion criteria	Patients who had not received early antibiotic treatment before lumber puncture	
	Uncentrifuged CSF specimens with adequate residual volume of ≥500 μL	
Patients exclusion criteria	Repeated specimens from the same patient	
**CSF inclusion criteria**		
Turbid appearance	The normal CSF should be clear and colourless, like ‘rock water’	[1]
Leukocytosis	> 5 × 10^6^ cells/L, or >15 × 10^6^ cells/L for infants aged <30 days	[7]
Polymorphonuclear cell predominance	> 50% of leukocytes	[7]
Abnormal chemistry results	Glucose <40 mg/dL, or protein > 1g/L	[2]

Abbreviation: CSF, cerebrospinal fluid.

CSF samples obtained via lumbar puncture were transported to the laboratory of Medical Microbiology in a cool box within 1 h. Gram staining was performed with cytocentrifuge specimens. CSF specimens with inclusion criteria described in Table 1 were cultured on blood agar, chocolate agar and MacConkey agar (Merck, Germany). The plates were incubated aerobically in a carbon dioxide enriched atmosphere at 35–37°C for 24–48 h. The identification of bacterial colonies was carried out using Gram staining and standard biochemical tests. The remaining CSF samples were preserved at −70°C for molecular analysis.

### Reference strains

The following reference strains were used as positive controls: *Streptococcus pneumoniae* ATCC49619, *Haemophilus influenzae* ATCC33930, *Neisseria meningitidis* ATCC10377, *Listeria monocytogenes* ATCC49594, *Streptococcus agalactiae* ATCC13813 and *Escherichia coli* ATCC35218.

### DNA extraction

Extraction of DNA from CSF samples was performed according to the protocol provided with the QIAGEN DNA Mini kit (QIAGEN Inc., Valencia, CA). To facilitate full lysis of Gram-positive bacteria, a modified protocol was used. Briefly, 200 μL of CSF was added to100 μL of sterile TE buffer (Tris- EDTA) and 0.5 μL lysozyme suspension (100 mg/mL) and incubated at 37°C for 1 h.

All subsequent steps were performed according to the kit protocol. The concentration and purity of DNA samples were determined using a NanoDrop Spectrophotometer (ND-1000; NanoDrop Technologies, Wilmington, DE) at 260 and 260/280 nm, respectively.

### PCR amplification

PCR was done for CSF specimens that met the criteria for culture. For detection of bacterial agents by PCR, the *ply* gene of *S. pneumoniae*, *ctrA* gene of *N. meningitidis*, *bexA* gene of *H. influenza*, *prf* gene of *L. monocytogenes, meth* gene of *E. coli* and *cyl* gene of *S. agalactiae* were used as species-specific targets. The oligonucleotide primers used in this study are listed in Table 2. Simplex PCR was performed using DreamTaq PCR Master Mix (Thermo Fisher Scientific), which contains Taq polymerase, dNTPs, MgCl2 and the appropriate buffer. Each PCR tube contained 25 μL reaction mixture composed of 12.5 μL of the master mix, 1.5 μL of each forward and reverse primer solution (in a final concentration of 200 nM), 3 μL of DNA and nuclease-free water to complete the final volume. PCR was performed using the Gene Atlas 322 system (ASTEC). Amplification involved an initial denaturation at 94°C, 5 min followed by 35 cycles of denaturation (94°C, 1 min), annealing (60°C, 1 min) and extension (72°C, 1 min), with a final extension step (72°C, 10 min). The amplified DNA was separated by submarine gel electrophoresis, stained with ethidium bromide and visualized under UV transillumination.

**Table 2:** Primers used in this study

Target	Primer sequence (5′→3′)	Amplicon size (bp)	Reference
*Nisseria meningitidis (ctrA)*	F: GCTGCGGTAGGTGGTTCAA R: TTGTCGCGGATTTGCAACTA	110	[23]
*Haemophilus influenza (bexA)*	F: TGCAGAGCGTCCTTTGGTCTAT R: CTCTTACTCGTGGTTTCCAACTTGA	114	[23]
*Streptococcus pneumonia (ply)*	F: GGCGAAATGGTGCTGGTAA R:GCCAAGAGATACTCATAGAACGTT	80	[23]
*Listeria monocytogenes (Prf)*	F: TCATCGACGGCAACCTCGG R: TGAGCAACGTATCCTCCAGAGT	114	[24]
*Escherichia coli (meth)*	F: GCTGCGGTGGTATGGTTCAA R: CTCTTACTCTTGGTGTCCAACTTGA	106	[25]
*Streptococcus agalactiae (cyl)*	F: TTTCACC AGCTGTATTA GAATA R: GTTCCCTGAACATTATCTTTGAT	263	[26]

### Statistical analysis

The data were analysed with SPSS version 17.0 software (SPSS, Inc., Chicago, IL). The Wilcoxon-Mann–Whitney and chi-square tests were used to determine the statistical significance of the data. A p < 0.05 was considered significant.

## Results

**Fig. 1:**
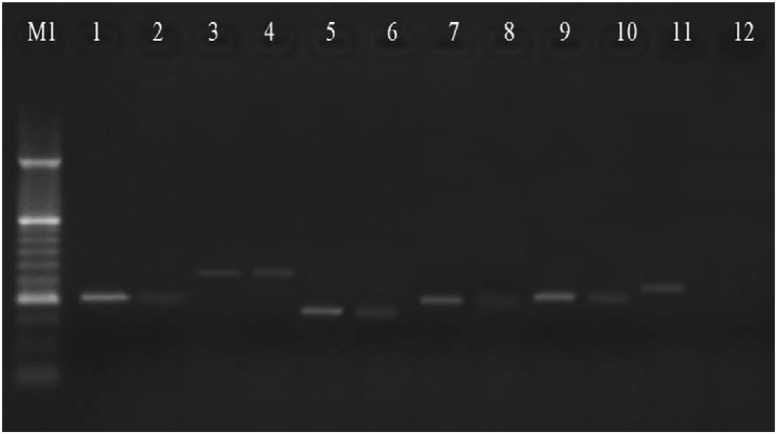
Agarose gel electrophoresis of PCR products. Lane 1: *Nisseria meningitidis (ctrA)* control positive strain. Lane 2: Positive clinical sample for *Nisseria meningitides*. Lane 3: *Streptococcus agalactiae (cyl)* control positive strain. Lane 4: Positive clinical sample for *Streptococcus agalactiae*. Lane 5: *Streptococcus pneumonia (ply)* control positive strain. Lane 6: Positive clinical sample for *Streptococcus pneumonia*. Lane 7: *Escherichia coli (meth)* control positive strain. Lane 8: Positive clinical sample for *Escherichia coli*. Lane 9: Positive clinical sample for *Listeria monocytogenes(Prf)*. Lane 10: Positive clinical sample for *Listeria monocytogenes*. Lane 11: *Haemophilus influenza (bexA)* control positive strain. Lane 12: Negative clinical sample.

A total of 100 CSF samples were collected from suspected cases of bacterial meningitis aged from 1 day to 78 years. Among the total patients, 52% of patients were aged <1 year, 24% were aged 1–10 years, 13% were aged 10–30 years and 11% were aged >30 years. The sex distribution was 52% male and 48% female. Demographic, clinical and laboratory characteristics of patients are shown in Table 3, Table 4. Fever, leukocytosis, headache, nausea and vomiting were the most common clinical manifestations of bacterial meningitis in our study. We also found that the leucocyte count, glucose and protein levels in CSF samples were strongly associated with bacterial meningitis. Forty-six percent of CSF samples had positive bacterial culture, of which 25 isolates (54.3%) were Gram-positive cocci and 21 (45.6%) were also Gram-negative bacilli. However, molecular analysis indicated that 52% of CSF samples were PCR positive. Also, 46% of CSF samples were positive by both culture and PCR, and 6% were positive only by PCR. Of 52 patients with bacterial meningitis, 30 patients (57.7%) were aged <1 year, ten patients (19.2%) were aged 1–10 years, six patients (11.5%) were aged 10–30 years and six patients (11.5%) were aged >30 years. The distribution of bacterial pathogens in CSF samples is shown in Table 3 and Fig. 1. The most prevalent pathogen was *S. pneumoniae* (36.5%), followed by *N. meningitidis* (28.8%) and *S. agalactiae* (15.4%). Table 4 demonstrates that the main cause of bacterial meningitis was distinct in different age groups. While in patients aged <1 year, *S. pneumonia, N. meningitidis* and group B streptococcus were the most common pathogens causing bacterial meningitis, and in patients aged between 1 and 10 years, *E. coli* was the most common.

**Table 3:** Demographic, clinical, and laboratory characteristics of patients

Demographic and clinical characteristics	Patients (n = 100)
Male (%)	52
Median age in years (range)	10.8 (0–78)
Admitted to intensive care unit (%)	49
Admitted to paediatrics unit (%)	39
Admitted to neurology unit (%)	12
Altered mental status on admission (%)	35
**CSF cell counts and chemistry**	
Leukocyte count (%)	
<5 × 10^6^ cells/L	47
10–99 × 10^6^ cells/L	30
>100 × 10^6^ cells/L	23
>100 mg/dL protein (%)	53
<100 mg/dL protein (%)	47
<40 mg/dL glucose (%)	51
>40 mg/dL glucose (%)	49
**Gram stain with any bacteria (%)**	48
**Culture results**	
Positive for any bacteria (%)	46
*S. pneumoniae* (%)	18
*N. meningitidis* (%)	13
*H. influenzae* (%)	3
*Streptococcus agalactiae* (%)	7
*Listeria monocytogenes* (%)	0
*Escherichia coli* (%)	5
**PCR results**	
Positive for any bacteria (%)	52
*S. pneumoniae* (%)	19
*N. meningitidis* (%)	15
*H. influenzae* (%)	4
*Streptococcus agalactiae* (%)	8
*Listeria monocytogenes* (%)	1
*Escherichia coli* (%)	5

Abbreviation: CSF, cerebrospinal fluid.

**Table 4:** Age stratification of bacterial meningitis patients based on PCR

Bacterial agents		Age (years)				Total
		<1	1–10	10–30	>30	
*S. pneumoniae*	Culture positive	11	3	2	2	18
	PCR positive	11	3	3	2	19
*N. meningitidis*	Culture positive	10	1	2	0	13
	PCR positive	10	2	2	1	15
*S. agalactiae*	Culture positive	7	0	0	0	7
	PCR positive	8	0	0	0	8
*E. coli*	Culture positive	0	5	0	0	5
	PCR positive	0	5	0	0	5
*L. monocytogenes*	Culture positive	0	0	0	0	0
	PCR positive	1	0	0	0	1
*H. influenzae*	Culture positive	0	0	1	2	3
	PCR positive	0	0	1	3	4
Total	Culture positive	28	9	5	4	46
	PCR positive	30	10	6	6	52

## Discussion

Fast and accurate diagnosis of bacterial meningitis, as a life-threatening disease, is very important for effective antimicrobial therapy [8]. The PCR is a sensitive and rapid method to detect bacterial pathogens that are difficult to culture [9]. We evaluated culture and species-specific PCR assay for the diagnosis of bacterial meningitis. According to our results, PCR (52%) had a higher positive rate than CSF culture (46%; p > 0.05), and it showed a good correlation between culture and PCR. Sacchi et al. reported that the presence of antibiotic activity in CSF is a strong risk factor for a real-time PCR-positive, culture-negative case. According to those findings, the real-time PCR might be particularly useful in settings where patients often receive antibiotics before lumbar puncture [[11], [12], [13]]. PCR assays do not require viable bacteria for positive results and are generally considered to be highly sensitive [2]. However, the presence of PCR inhibitors in clinical specimens such as IgG in blood samples can reduce the amplification efficacy of PCR and might explain the imperfect PCR sensitivity in this and other studies [7,14]. Similar to Nhantumbo et al. report, we found that a significant number of CSF samples (48%) were negative for the investigated bacteria [12]. This likely suggests that viruses, parasites and other bacterial agents might be causing meningitis.

In our study, the most prevalent pathogen was *S. pneumoniae* (36.5%), followed by *N. meningitidis* (28.8%) and *S. agalactiae* (15.4%). This finding is in accordance with studies performed in developing countries where meningitis vaccines are not administered [15]. In the recent decade, *S. pneumoniae* has been considered as the main causative agent of bacterial meningitis in children aged <10 years, in elder population, and in immunocompromised patients in the United States and European countries [16]. According to previous studies, the frequency of *S. pneumoniae* was 31.7–73.8% [12,12, 17]. This variation in *S. pneumoniae* frequency may be because of the performance of pneumococcal vaccination programme in some countries, patients’ age, diagnostic approaches used, type of specimens and etc. According to Houri et al. meta-analysis, the frequency of pneumococcal meningitis in children aged <10 years and individuals aged >10 years in Iran was 36% and 20%, respectively [17]. The high prevalence of pneumococcal meningitis in Iran is alarming since the emergence of drug-resistant strains and unavailability of the 7-valent pneumococcal conjugate vaccine in this region. Therefore, pneumococcal meningitis could have the potential to become a serious life-threatening infection in Iran.

*N. meningitidis* is the second most common cause of bacterial meningitis in North America, with an incidence of 0.6 per 100 000 populations. In studies conducted in the United Kingdom, investigators have dramatically increased the sensitivity of diagnosis with PCR assay. They have been able to confirm 56% more cases of invasive meningococcal disease with PCR than with culture [4]. According to our results, *N. meningitidis* (28.8%) was the second most common causative agent of bacterial meningitis. In accordance with our finding, a report from Turkey showed that *N. meningitidis* serogroup W135 was the dominant bacterial agent of meningitis in children [18]. However, the prevalence of meningococcal meningitis in Iran was reported with lower frequency and accounts for 13% of all cases [17].

In previous reports of Iran, Hib (15%) was the second most common causative agent of bacterial meningitis [17]. Based on the CDC reports in 2004, the rate of Hib meningitis was <15 per 100,000 in Iran [19]. After the introduction of the DTPw-HB/Hib vaccine in 2014, a significant reduction in Hib meningitis was reported in Iran [17].

According to our results, the frequency of *H. influenzae* (7.7%) was lower than studies conducted before 2014. However, molecular typing of *H. influenza* was not performed, and for this reason, we were not able to discriminate type b and non–type b strains of *H. influenzae.*

In many countries, especially in Sub-Saharan Africa and Asia, *S. agalactiae* is an important cause of bacterial meningitis in the neonates [12]. In many countries, *S*. *agalactiae* is a predominant agent of neonatal meningitis with high morbidity and mortality [12]. Our finding showed that *S. agalactiae* (15.4%) was the third most common causative agent of meningitis. All eight patients with group B streptococcus meningitis were aged <1 year.

*L. monocytogenes* is the third most common cause of bacterial meningitis in immunocompromised patients, elderly individuals and accounts for approximately 20% of neonate's meningitis [20]. In our study, *L. monocytogenes* was not isolated from CSF culture, and PCR assay showed that only one CSF sample was PCR positive. This patient with PCR positive for *L. monocytogenes* was aged <1 year. This finding is in agreement with the previous reports on the isolation of *L. monocytogenes* in Iran [21]. It is not clearly known whether *L. monocytogenes* is not truly prevalent in our country or this is because of the lack of standard techniques required for its isolation.

Gram-negative enteric bacilli usually cause meningitis after head trauma or neurosurgery and are causative agents of community-acquired meningitis. In previous reports from Iran, Enterobacteriaceae was a common etiological agent of acute bacterial meningitis [22]. In our study, the frequency of *E. coli* in PCR-positive CSF samples was 9.6%. All patients with *E. coli* meningitis were aged 1–10 years.

## Conclusion

Our findings in agreement with other reports from Iran indicate that the most common causative agents of bacterial meningitis in our country are vaccine-preventable pathogens. Therefore, the prevention and control measures should be considered to reduce the incidence of bacterial meningitis. According to our results, PCR assay is a more accurate and rapid diagnostic tool for bacterial meningitis. So, the clinical application of this technique may help to reduce the potential risk of delay in antimicrobial therapy of meningitis.

## Transparency declaration

The authors declare that they have no competing interests.
